# Terahertz Wave Absorber Relying on Strontium Titanate and Dirac Semimetal for Dual Adjustability

**DOI:** 10.3390/mi17020266

**Published:** 2026-02-20

**Authors:** Zeng Qu, Mengyuan Zhao, Yuanhao Huang, Yibin Gong, Shishengdian Lu, Xuanqi Zhang, Jiayun Wang, Yuanhui Wang, Yinuo Cheng, Binzhen Zhang

**Affiliations:** 1 School of Electrical and Control Engineering, North University of China, Taiyuan 030051, China; 2 State Key Laboratory of Extreme Environment Optoelectronic Dynamic Measurement Technology and Instrument, Taiyuan 031151, China; 3 School of Instrument and Electronics, North University of China, Taiyuan 031151, China; 4 Glasgow College, University of Electronic Science and Technology of China, Chengdu 611731, China

**Keywords:** terahertz absorber, strontium titanate, Dirac semimetal

## Abstract

Limited by the material response characteristics and structural design, the development of dynamically tunable terahertz absorbers with multi-functional properties remains a major challenge. In this study, a dual-tunable terahertz absorber based on the synergistic integration of strontium titanate (STO) and Dirac semimetal (BDS) is proposed. By utilizing the temperature-sensitive dielectric constant of STO and the electrically tunable conductivity of BDS, the device can realize on-demand switching between a broadband absorption mode (absorptivity >90% in the 1.347~2.1271 THz band) and a dual-narrowband absorption mode under external field excitation. Notably, the centrosymmetric cross-patterned structure on the top layer ensures the polarization insensitivity of the device, and this single structure can also serve as a high-sensitivity temperature sensor. Simulation results verify that the device exhibits stable performance under different incident angles and environmental variations. This study constructs a compact multi-functional device platform integrating dynamic absorption regulation and in situ sensing, which provides a new technical route for the development of intelligent terahertz systems in the fields of terahertz imaging, communication, detection and other related areas.

## 1. Introduction

The terahertz band encompasses 0.1–10 THz electromagnetic oscillations, with vacuum wavelengths spanning 30 μm to 3 mm. These waves hold vast bandwidth resources, which can efficiently relieve the overcrowding in the existing communication frequency bands [1]. Terahertz radiation exhibits distinctive properties including exceptional penetration capability, material-selective attenuation, and non-ionizing photon characteristics. As high-speed optical sensors, terahertz metamaterial sensors show significant advantages in detection technology. Thanks to the characteristics of terahertz waves and the responsiveness of metamaterial devices to variations in the surrounding dielectric environment, these sensors are capable of providing high sensitivity, high efficiency, and non-destructive measurements. THz material-based sensing demonstrates transformative prospects across biomedical diagnostics, industrial NDT (Non-Destructive Testing), and agricultural product quality monitoring [2]. Rapid progress in these fields has recently stimulated extensive research on THz-based devices [3,4,5], including filters, reflectors, absorbers, and lenses. Metamaterials are a widely used approach to enhance the sensitivity of terahertz sensing [6]. Terahertz metamaterial perfect absorbers, capable of fully absorbing incoming electromagnetic waves without any reflection or transmission at certain frequency points or ranges, have generated significant interest among researchers.

In recent years, the design of terahertz absorbers has become a research hotspot in the terahertz field [7,8]. Among them, dielectric absorption materials have attracted extensive attention due to their prominent application potential. Based on various metamaterial systems, researchers have designed terahertz absorbers with different absorption frequencies, efficiencies and peak numbers [9,10]. However, such absorbers have an inherent defect of passive operation, whose optical functions are fixed after fabrication and cannot dynamically regulate the absorption frequency and efficiency [11,12]. Researchers have also conducted studies to address this issue. In terms of external field tuning, Zhou Zhen et al. proposed a LFO/fluorophlogopite bilayer structure to realize magneto-laser dual modulation of terahertz response [13]. The CFO/magnesium oxide/fluorophlogopite three-layer composite structure designed by this team can achieve nonlinear suppression of terahertz transmittance under a magnetic field of 0~300 millitesla, with a modulation depth of 13% [14]. Huang Xin et al. combined graphene with strontium titanate to realize narrowband absorption in different frequency bands through chemical potential and temperature regulation [15]. As a three-dimensional structural analog of graphene [16], Dirac semimetal (BDS) has emerged as a new entry point for the design of tunable terahertz absorbers due to its structural stability, high electron mobility, and low intrinsic loss [17], and the continuous tunability of its electrical conductivity in the terahertz band via chemical doping or external voltage application [18]. However, most of the currently designed terahertz absorption devices based on BDS only have a single regulation mode and lack flexible multi-dimensional regulation means. For example, in 2020, Xiong et al. proposed a bifunctional terahertz absorber composed of graphene and BDS, which can realize independent or combined tuning of absorption bandwidth by regulating the Fermi levels of graphene and BDS separately or synergistically [19]. In the same year, the BDS–insulator–metal (BIM) three-layer stacked structure designed by Shi et al. can realize the frequency tunability of perfect absorption peaks by regulating the Fermi level of BDS [20]. As a ferroelectric material, strontium titanate (STO) has both a high dielectric constant and low dielectric loss, and its relative permittivity can be effectively regulated by temperature, which provides advantages for terahertz thermal frequency regulation [21]. Based on the electrical tunability of BDS and the thermal tunability of STO, the combination of the two to realize independent and synergistic regulation of absorption characteristics has become a new design idea in this field. Shen Qiang et al. realized dual-harmonic tuning of terahertz absorbers based on the characteristics of Dirac semimetal and strontium titanate, achieving dual regulation of the center frequency and amplitude of narrowband waves [22]. The team led by Wu Tong developed a broadband terahertz absorber with dynamically adjustable spectral coverage by using Dirac semimetal and strontium titanate materials; this design can maintain stable broadband absorption characteristics for polarized waves within an incident angle of 40° [23]. Existing studies mostly focus on the regulation of a single dimension (narrowband or broadband), while the dynamic switching combination of narrowband and broadband absorption can further expand the application range of devices and improve application flexibility.

In this paper, a multi-tunable composite terahertz metamaterial absorber based on crystalline STO and BDS is proposed, which integrates the Fermi level-sensitive conductivity of BDS and the temperature-tunable dielectric property of STO. This design enables the device to realize dynamic tuning of absorption performance while possessing temperature response characteristics and a dual-absorption mode switching function. The wave absorption mechanism of the absorber is explained through electric field distribution analysis and impedance matching theory, and its performance under different polarization states and incident angles is investigated. The absorber features a novel design, a wide tuning range, good temperature responsiveness and switchable dual modes, thus exhibiting important application potential in terahertz systems.

## 2. Working Principle and Structural Design

**Figure 1:**
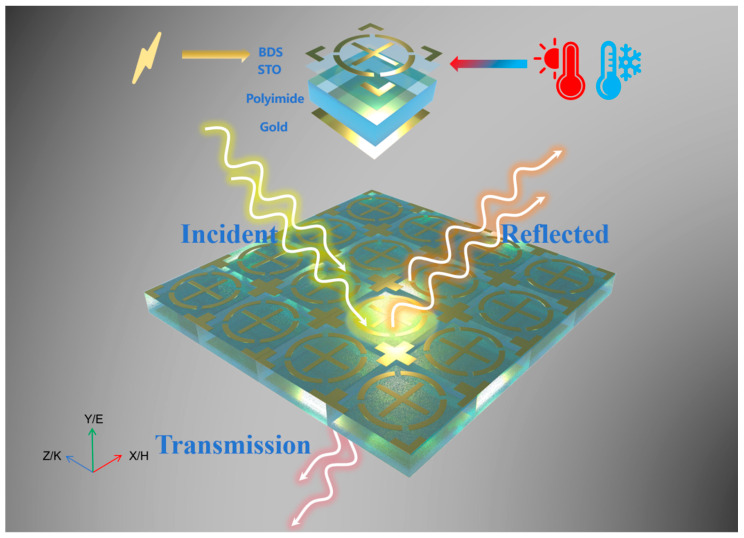
Principle and structure of dual-tunable temperature sensor.

**Figure 2:**
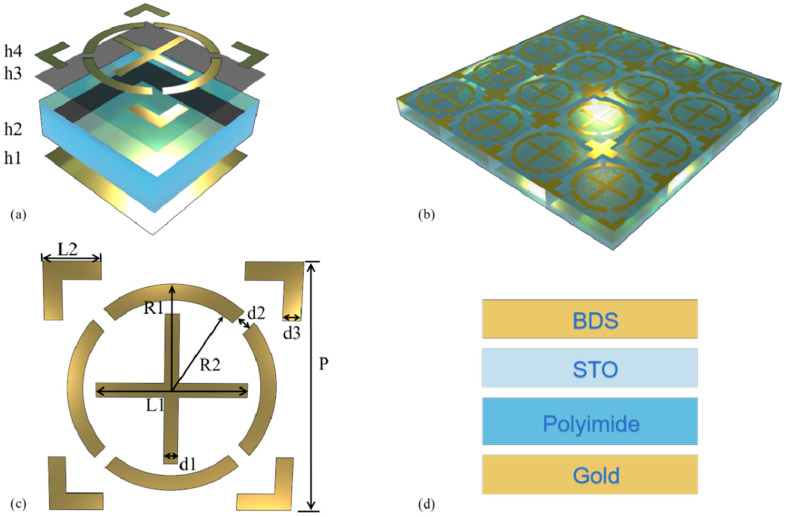
Configuration schematic of the THz sensor unit. (**a**) Three-dimensional side view of the device. (**b**) Overall view of the device. (**c**) Surface layer perspective. (**d**) Structural material diagram.

A terahertz absorber with dynamic tunability via the Fermi level and temperature is designed in this paper. Figure 1 illustrates the function and structure of the device. Figure 2 shows the structural schematic of the dual-tunable terahertz absorber, with the design principles as follows: The top layer of the absorber adopts a centrosymmetric Dirac semimetal (BDS) pattern, which achieves TE/TM polarization insensitivity based on symmetric design; this structure also relies on the Fermi level response characteristic of Dirac semimetals to realize efficient switching between two absorption modes. The second layer is a temperature-sensitive STO film, which endows the device with temperature sensing capability by utilizing the characteristic of its dielectric constant changing dynamically with temperature. The third layer is a polyimide-based polymer dielectric layer with a dielectric constant of 3.1 and a loss tangent of 0.02; its thickness (17 μm) is determined via simulation optimization to balance dielectric loss and absorption bandwidth and ensure high absorption efficiency. The bottom layer is a metal reflective layer to suppress transmission, and its performance is optimized based on impedance matching theory to maximize absorption efficiency. The synergistic design of the BDS pattern and STO layer enables the integration of dual functions: Fermi level-regulated mode switching + temperature-regulated frequency shift. Table 1 lists all structural parameters of the device in detail, and these parameters are all obtained through systematic simulation optimization to achieve the optimal absorption performance and tunable range.

In the simulation of temperature sensors based on STO and BDS, a periodic unit pattern was applied to the x-y plane, and open boundary conditions were set in the z-direction to simulate real-world environments.

BDS architectures permit Fermi level control via external voltage biasing. Combined with the temperature-dependent dielectric constant of STO, this enables dynamic switching of the terahertz wave absorption characteristics:

The dielectric constant of Strontium titanate, a material responsive to temperature fluctuations, varies with the changing environmental temperature. The theoretical value of the dielectric constant can be expressed using the following formula [24,25]:

(1) $$\varepsilon_{\omega }=\varepsilon_{\infty }+\frac{\alpha }{{\omega_{0}}^{2}+\omega^{2}-i\omega \gamma }$$

In Equation (1), $\varepsilon_{\infty }$ represents the dielectric constant at high frequencies, measuring 9.6; α represents the oscillator strength, unaffected by temperature, with a magnitude of $2.3\times 10^{6}{\mathrm{cm}}^{2}$; ω is the angular frequency; $\omega_{0}$ denotes the soft-mode frequency adjusted via Cochran’s law; γ denotes the damping parameter; $\omega_{0}$ and both γ and can be calculated using the following formula [26]:

(2) $$\omega_{0}(T)[{\mathrm{cm}}^{-1}]=\sqrt{31.2(T-42.5)}$$

(3) $$\gamma (T)[{\mathrm{cm}}^{-1}]=-3.3+0.094T$$

**Figure 3:**
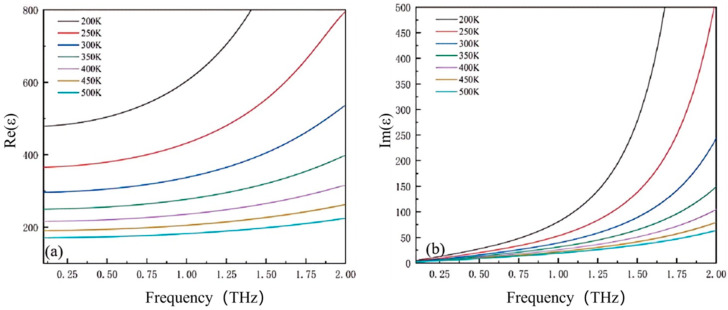
Dielectric constant of strontium titanate at different temperatures: (**a**) real part; (**b**) imaginary part.

T denotes temperature (in K), which is a key factor affecting the parameter variations in Equations (2) and (3). Figure 3 reveals strong thermal sensitivity in strontium titanate’s dielectric behavior: Real permittivity experiences a monotonic decline with rising temperature, whereas imaginary permittivity displays an inverse correlation. Crucially, the attenuated fluctuation in loss component (<5% deviation) versus substantial real permittivity variation enables absorber stability under thermal cycling (ΔT = 200 K).

**Figure 4:**
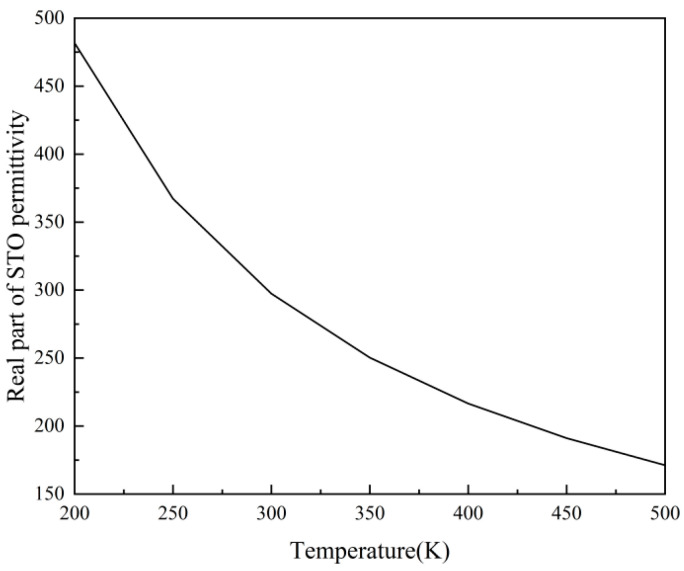
Temperature-dependent real permittivity of SrTiO_3_.

Figure 4 offers a more straightforward and easily grasped depiction of how the dielectric function of STO material changes with temperature. As the temperature steadily rises from 200 K to 500 K, the real component of the dielectric function experiences a decline, dropping from 481.70978 to 171.16768. The dielectric function of STO does not exhibit a linear relationship with temperature; rather, at lower temperatures, the curve drops more steeply, and the rate of change becomes greater.

The random phase approximation method serves as the theoretical foundation for this analysis [27], the dielectric permittivity of Dirac semimetals at terahertz frequencies is given by [28]

(4) Re(σ(Ω))=e2h¯gkF24πΩG(Ω2)

(5) $$I_{m}(\sigma (\Omega ))=\frac{e^{2}}{\bar{h}}\frac{gk_{F}}{24\pi }\left\{\frac{4}{\Omega }\left[1+\frac{\pi^{2}}{3}{\left(\frac{T}{E_{F}}\right)}^{2}\right]\right.+\left.8\Omega \int_{0}^{\varepsilon_{c}}\left(\frac{G(\varepsilon )-G(\frac{\Omega }{2})}{\Omega^{2}-\varepsilon^{2}}\right)\varepsilon d\varepsilon \right\}$$

In Equations (4) and (5), $G(E)=n(-E)-n(E)=\sinh(E/T)/[\cosh(E_{F}/T)]$, *n(E)* denotes the Fermi–Dirac distribution. The configuration degeneracy is quantified as *g* = 40, $k_{F}=E_{F}/\bar{h}v_{F}$ is the Fermi momentum; *T* denotes a non-zero temperature; *ħ* is the reduced Planck constant; $v_{F}$ represents the Fermi velocity, whose value is $10^{6}$m/S; $E_{F}$ denotes the Fermi energy level. The analysis indicates that the electrical conductivity of Dirac semimetal is primarily determined by the Fermi energy .μ represents carrier mobility, with a value of $6.42\times 10^{4}$ cm^2^V^−1^S^−1^; $\Omega =\bar{h}\omega /E_{F}+Fjv/(E_{F}k_{F}v)$ is the scattering rate; $\varepsilon_{c}=E_{c}/E_{F}$, $\varepsilon_{c}$ denotes the cutoff energy, whose value is 3 [29].

In the simulations of this paper, we selected key parameters of Dirac semimetals, including carrier mobility, Fermi velocity, and cutoff energy. These parameters exhibit variability due to the influence of multiple factors such as material quality, defects, impurities, and strain. However, the parameters adopted in the simulations represent the typical values of high-quality Dirac semimetals. Moreover, based on the consideration of the variation range in practical applications, the reliability and universality of the simulation results have been ensured [30].

**Figure 5:**
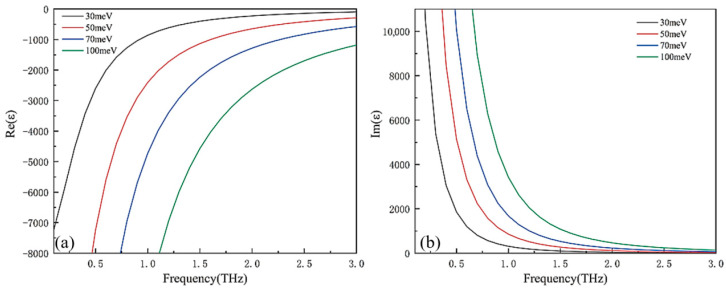
Dirac semimetal permittivity vs. Fermi energy. (**a**) Real component. (**b**) Loss component.

Because the dielectric constant is intrinsically linked to conductivity, adjusting the Fermi energy level offers a direct way to fine-tune a material’s dielectric properties. As illustrated in Figure 5, an upward trend in the Fermi energy level leads to a steady decline in the real component of the dielectric constant, while its imaginary counterpart sees a corresponding rise. This interplay is crucial for optimizing wave absorption capabilities by strategically manipulating the Fermi energy level.

Owing to the extremely fast relaxation rate of Dirac semimetals, the electrical modulation switching speed reaches the picosecond level, which meets the demand for high-speed tunability. A back-gate structure gating design is adopted for Fermi level adjustment. Based on the experimental carrier concentration, the Fermi level can be tuned simply by adjusting the voltage under low-energy conditions.

This paper employs CST Studio Suite 2023 for the simulations. Since the absorption rate cannot be directly obtained from the simulations, an absorption formula needs to be introduced:

(6) $$A=1-|S_{11}{|}^{2}-|S_{21}{|}^{2}$$

Absorption efficiency is governed by $S_{11}$ and $S_{21}$ coefficients as per wave optics principles. The implemented structure features a subwavelength metal ground plane, effectively nullifying transmission. Consequently, the absorptivity simplifies to $A=1-{\left|S_{11}\right|}^{2}$ [31].

To better position the temperature sensors, it is essential to conduct an in-depth investigation into the wave absorption principle at the sensor location. The absorber’s operational principle is governed by impedance matching theory [32], with its normalized impedance derived from:

(7) $$Z=\sqrt{\frac{\mu }{\varepsilon }}=\sqrt{\frac{{\left(1+S_{11}\right)}^{2}-{S_{21}}^{2}}{{\left(1-S_{11}\right)}^{2}-{S_{21}}^{2}}}$$

(8) $$R={\left|\frac{Z-Z_{0}}{Z+Z_{0}}\right|}^{2}$$

(9) A=1−R

The normalized impedance (Z), wave impedance (R), and absorptivity (A) of the THz device are governed by Equation (1), with μ, ε, and $Z_{0}$ denoting permeability, permittivity, and free-space impedance respectively. This formulation reveals that impedance engineering minimizes THz wave reflection through internal energy dissipation. Optimal absorption occurs when structural impedance matches $Z_{0}$, driving the reflection coefficient to zero.

## 3. Simulation Results and Discussion

**Figure 6:**
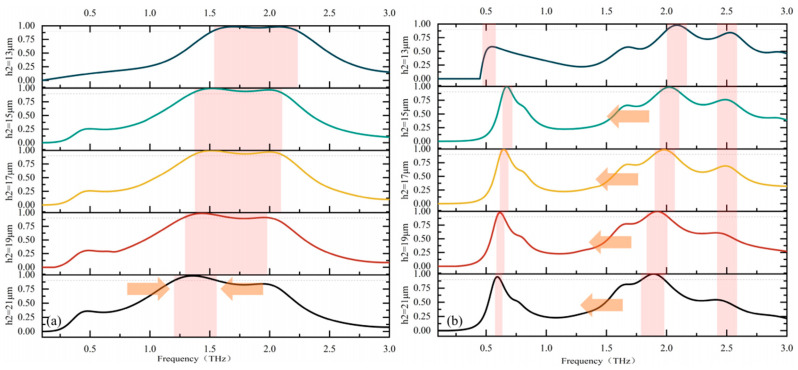
Dielectric thickness-dependent absorption tuning: (**a**) the effect on broadband absorption; (**b**) the effect on narrowband absorption.

The subsequent analysis examines the impact of the polyimide layer’s thickness within the dielectric layer on the absorber’s performance. The analysis of these parameters enables us to ascertain the optimal dimensions. As shown in Figure 6, we investigated the fluctuation of broadband and narrowband absorptivity within the dielectric layer thickness range of 13 μm to 21 μm, and the different curves in the figure reflect these variations. When considering broadband absorption, it can be observed that an increase in the dielectric layer thickness leads to a corresponding reduction in the effective absorption range of the absorber, with the frequency range shifting gradually towards the red end. However, as the bandwidth redshifts, the two peak absorptivity values also change continuously. A thicker dielectric layer implies higher dielectric loss, which in turn increases the quality factor (Q-factor), resulting in higher peak absorptivity but a narrower bandwidth. In the case of narrowband absorption, as the thickness increases, the two peak frequencies move closer to each other, and the amplitudes of the two peaks exhibit opposite trends: the first absorption peak decreases slightly while the second one increases slightly. In addition, it can be seen that the structural parameters also affect the emergence of the third peak near the 3 THz side in narrowband absorption, providing a feasible approach for the realization of multi-band absorption. To achieve wider bandwidth absorption while maintaining high absorptivity, we selected 17 μm as the optimized parameter.

**Figure 7:**
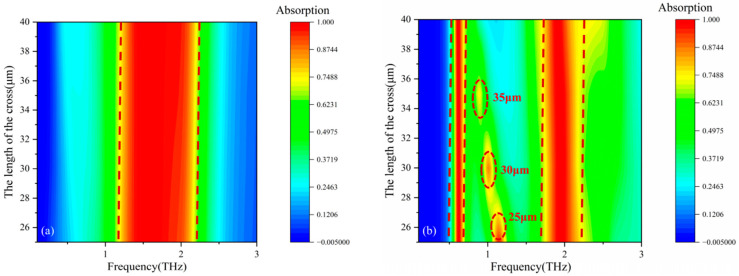
Effect of internal cross structure length on absorption: (**a**) effect on broadband absorption; (**b**) effect on narrowband absorption.

To further investigate the impact of other structures on the absorber, the following figure illustrates how the absorption rate of the device is affected by internal cross structures of varying lengths. As shown in Figure 7a, the bandwidth of the broadband absorption band is nearly unaffected by this structural variation, with only minor changes observed in the absorption rate at the second peak. In contrast, for the narrowband absorption depicted in Figure 7b, the first absorption peak and the peak near 3 THz remain largely unchanged, while other resonant peaks between them are influenced. Specifically, when the cross length is set to 25 μm, 30 μm, and 35 μm, the peak values of the interfering resonant peaks gradually decrease, and the interfering resonant peak eventually disappears at 40 μm. To achieve broader bandwidth absorption with reduced interference and higher absorption efficiency, we select 40 μm as the optimized parameter.

**Figure 8:**
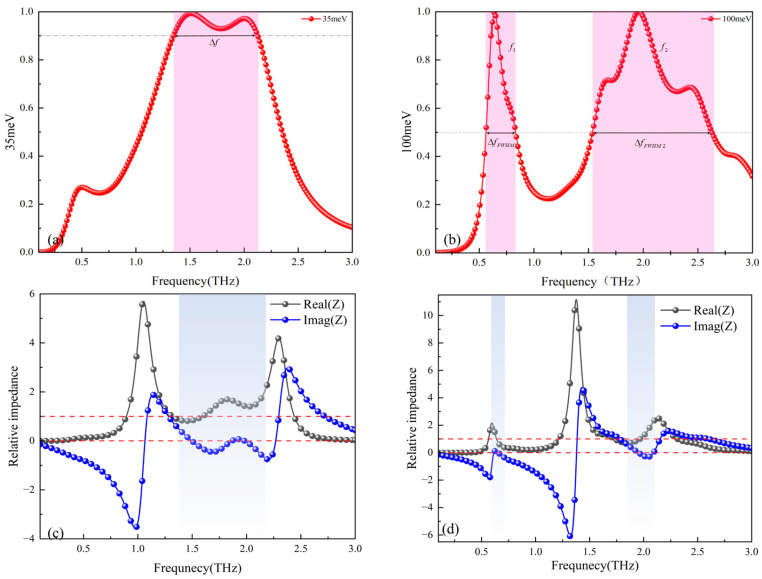
(**a**) Wave absorption curve at a Fermi level of 35 meV; (**b**) wave absorption curve at a Fermi level of 100 meV; (**c**) equivalent impedance at a Fermi level of 35 meV; (**d**) equivalent impedance at a Fermi level of 100 meV.

Figure 8 shows the absorption spectra and equivalent impedances obtained using CST. Figure 8a and Figure 8b respectively display the absorption curves of the Dirac semimetal at an initial temperature of 200 K and Fermi levels of 35 meV and 100 meV. It can be observed from the figures that when the Fermi level is 35 meV, the broadband absorption rate exceeds 90% within the range of 1.347–2.1271 THz. The absolute bandwidth at this point is $f_{\Delta }=2.1271-1.347=0.7801THz$, the center frequency is $f_{c}=1.737THz$, and the relative bandwidth is $FBW=\frac{0.7801}{1.737}\times 100\%=44.9\%$.

When the Fermi level is 100 meV, two narrowband absorption peaks appear at 0.6394 THz and 1.9589 THz, with absorption rates of both peaks exceeding 90%, specifically 99.63% and 99.91% respectively. At this point, the full widths at half-maximum of the two absorption peaks are $\Delta f_{FWHM1}=0.2726THz$ and $\Delta f_{FWHM2}=1.0933THz$ respectively, the quality factors Q are $Q_{1}=\frac{f_{1}}{\Delta f_{FWHM1}}=\frac{0.6394}{0.2726}=2.346$ and $Q_{2}=\frac{f_{2}}{\Delta f_{FWHM2}}=\frac{1.9589}{1.0933}=1.79$, respectively. Additionally, due to the symmetry of the structure, the TE and TM modes have the same absorption. Therefore, by controlling the Fermi level of the Dirac semimetal, the functionality of the terahertz absorber can be flexibly switched between a broadband and two narrowbands.

Analysis based on the impedance matching theory shows that when the equivalent impedance of the device matches the free-space impedance (set to 1 in this paper), the reflectivity is the lowest. Under these conditions, optimal absorption performance is achieved when the real component of the wave impedance is approximately unity, while its imaginary element is minimized. In the broadband absorption mode with a Fermi level of 35 meV, the variation trends of the real part (black line) and imaginary part (blue line) of the impedance shown in Figure 8c confirm this theory: within the frequency band where the absorption rate is higher than 90%, the real component approaches unity, while the imaginary component tends toward zero.

This study conducted further validation of the impedance matching theory, the equivalent impedance in the dual-narrowband absorption mode when the Fermi level of the Dirac semimetal is 100 meV was simulated, as demonstrated in Figure 8d. In Figure 8d, the solid black and blue traces correspond to the resistive and reactive components of the device impedance, respectively. The two absorption peaks exhibit absorptivities of 99.63% and 99.91%, respectively. Figure 8d confirms that in the dual-narrowband regime, both peaks maintain exceptionally high absorption efficiency. Correspondingly, the real parts of the equivalent impedance of the two absorption peaks are closer to 1, and the imaginary parts are closer to 0. On the other hand, when the Fermi levels of the Dirac semimetal are 35 meV and 100 meV, by comparing the equivalent impedance of the broadband absorption peak with that of the narrowband absorption mode, a similar pattern is also observed. Compared with the broadband absorption mode, the real part of the equivalent impedance in the narrowband absorption mode is closer to 1, and the imaginary part is closer to 0. This result further verifies the matching theory of equivalent impedance.

**Figure 9:**
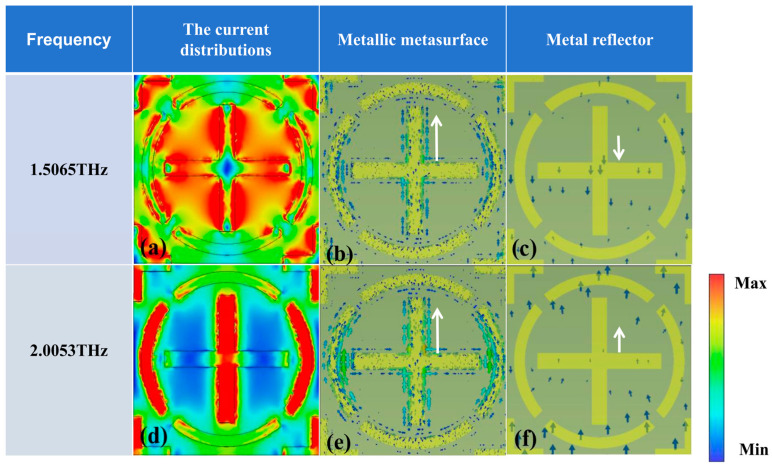
Electric field, Dirac semimetal surface, and metal reflector current distributions during broadband absorption: (**a**–**c**) show the current distributions of the electric field, metallic metasurface, and metal reflector at 1.5065 THz, respectively; (**d**–**f**) show the current distributions of the electric field, metallic metasurface, and metal reflector at 2.0053 THz, respectively.

To elucidate the physical origins governing these distinct absorption regimes, Figure 9 presents spatial electric field profiles for both broadband and dual-resonance operational states at their characteristic frequencies.

Figure 9a–f visualize electromagnetic responses across operational regimes, presenting spatial field profiles at 1.5065 THz and 2.0053 THz alongside current distributions on both Dirac semimetal surfaces and metallic backplanes. Panel (a) demonstrates pronounced field confinement along peripheral regions of the central cross-shaped element and adjacent split-ring resonators at 1.5065 THz. Complementary analysis of panel (d) reveals dominant field localization along the Y-oriented cross-member, gapped annular structures, and corner quarter-cross elements at 2.0053 THz. Current mappings in (b) and (c) exhibit antiparallel *Y*-axis conduction between the Dirac semimetal layer (bottom-to-top) and metallic ground plane (top-to-bottom) at 1.5065 THz. This counter-circulating charge transport establishes virtual current loops corresponding to magnetic dipole excitation, directly generating the 1.5065 THz resonance. Conversely, panels (e) and (f) depict co-directional *Y*-axis current alignment in both conductive layers at adjacent resonances (1.9589/2.0053 THz), with minor X-component circulation observed in the top layer. This fundamentally distinct behavior from the magnetic resonance regime indicates sustained Dirac plasmon excitation with intensified coupling efficiency, thereby producing the enhanced 2.0053 THz absorption peak.

**Figure 10:**
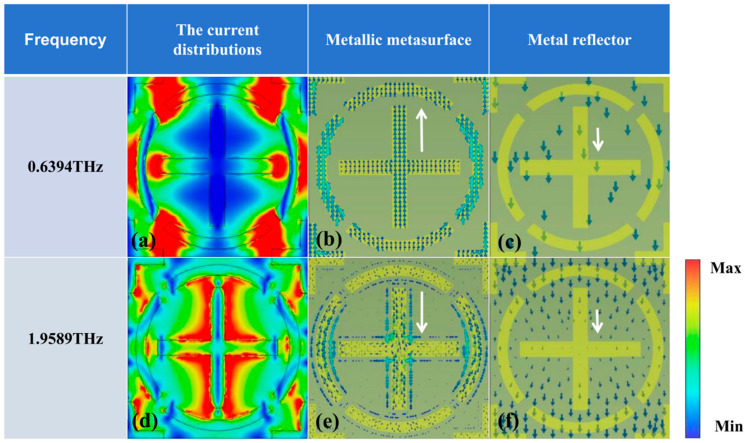
Current distribution diagrams of the electric field, Dirac semimetal surface, and metal reflective layer during narrowband absorption: (**a**–**c**) represent the current distributions of the electric field, metal metasurface, and metal reflective layer at 0.6394 THz, respectively; (**d**–**f**) represent the current distributions of the electric field, metal metasurface, and metal reflective layer at 1.9589 THz, respectively.

Electromagnetic responses at dual-resonance frequencies (0.6394 THz and 1.9589 THz) are visualized in Figure 10a–f, presenting field distributions and conductive layer charge transport mappings. Panel (a) reveals pronounced field confinement at split-ring apertures, external annular perimeters, and enclosed cross-termini during 0.6394 THz operation. Complementary panel (d) demonstrates dominant field localization around the Y-polarized cross-element at 1.9589 THz. Current analysis indicates antiparallel alignment between Dirac semimetal surface currents and metallic ground plane flows at the primary resonance (0.6394 THz), whereas co-directional charge transport occurs at the secondary resonance (1.9589 THz). This resonance dichotomy parallels broadband absorption behavior: magnetic dipole excitation generates the initial peak, while sustained plasmon excitation with intensified coupling produces the subsequent resonance enhancement.

It can be seen that the two peaks follow the same pattern in both broadband and narrowband absorption. This indicates that the broadband is formed by the superposition of multiple narrowband absorption peaks, which provides an idea for broadening the broadband range.

**Figure 11:**
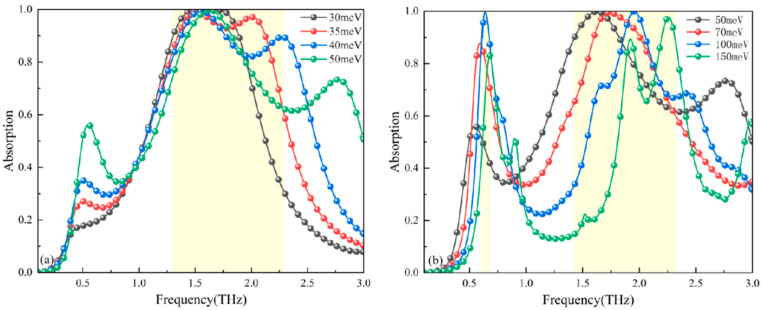
(**a**) Fermi level regulation during broadband absorption. (**b**) Fermi level regulation during narrowband absorption.

The operational mechanism establishes Fermi energy as a critical modulator of Dirac semimetal conductivity. Consequently, this analysis examines Fermi-level variations (30–50 meV) in BDS at fixed 200 K strontium titanate temperature, focusing on absorption characteristics during broadband operation (Figure 11a). Key observations reveal exceptional stability in the primary resonance peak: its frequency remains essentially invariant while intensity exhibits minimal fluctuation across the Fermi energy spectrum. Conversely, the secondary peak demonstrates significant spectral displacement toward higher frequencies with increasing Fermi energy, accompanied by moderate attenuation in absorptivity. Optimal broadband performance occurs at 35 meV, achieving >90% absorption across a 0.78 THz bandwidth. Spectral minima within the absorption band experience progressive degradation with Fermi-level adjustments, though maintaining >80% absorption efficiency within the 30–40 meV range.

Under elevated Fermi energies (50–150 meV) depicted in Figure 11b, distinct spectral modifications emerge. The primary absorption resonance maintains near-constant frequency comparable to broadband operation, though exhibiting substantial absorptivity magnitude variation. Conversely, the secondary peak undergoes progressive blue-shifting with increasing Fermi energy while retaining significant absorption efficiency. Collectively, these dual-resonance phenomena demonstrate pronounced Fermi-level dependence within the narrowband operational regime.

Some parameters of strontium titanate are modulated with the variation in the material’s temperature. Consequently, changes in the thermal environment of the detector unit induce corresponding modifications in the complex permittivity of strontium titanate. Since the spectral peak position is primarily determined by the real part of the permittivity, while the loss mechanism is dominated by the imaginary part of the permittivity, temperature variations ultimately result in resonant frequency shifts in the absorption characteristics.

**Figure 12:**
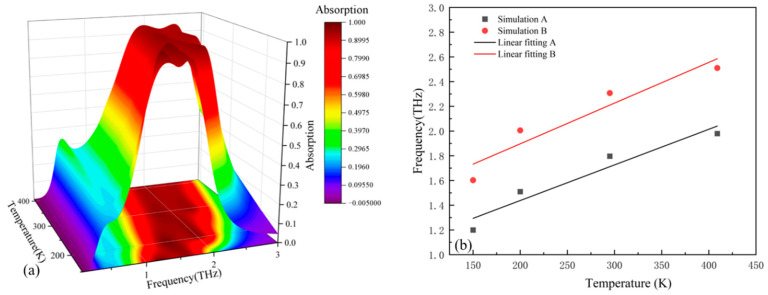
(**a**) Absorption spectra at different temperatures when the sensor is in the broadband absorption mode. (**b**) Center frequencies and fitted broken lines at different temperatures.

When the Fermi level of the Dirac semimetal is 35 meV, the absorption spectra of the sensor at different temperatures are shown in Figure 12a.

Temperature-dependent spectral shifts are demonstrated in Figure 12a: Peak I undergoes displacement from 1.2049 THz to 1.9792 THz across the 150–409 K thermal range, while Peak II migrates from 1.6022 THz to 2.5099 THz. The superposition of two adjacent resonant peaks forms a broadband. When the temperature changes, the variation in the dielectric constant of strontium titanate (STO) simultaneously modulates the resonant frequencies of the two peaks, causing them to shift synchronously, which in turn manifests as a shift in the center frequency of the entire broadband absorption band. The broadband absorption’s central frequency $f_{c}$ is mathematically defined as $f_{c}=(f_{-}+f_{+})/2$, where $f_{-}$ and $f_{+}$ denote the 90% absorptivity cutoff frequencies. Thermal progression induces simultaneous $f_{c}$ displacement from 1.386 THz to 2.2199 THz and absorption bandwidth expansion from 0.6119 THz to 0.8584 THz. During broadband operation, spectral position correlates with strontium titanate’s real permittivity component, whereas dissipation mechanisms primarily respond to its imaginary permittivity constituent.

Therefore, when the temperature of the device changes, the center frequency and absorption bandwidth of the broadband absorption peak will shift and widen respectively. Therefore, when the sensor is in the broadband absorption mode, a change in the device temperature can cause the center frequency and bandwidth to change accordingly.

**Figure 13:**
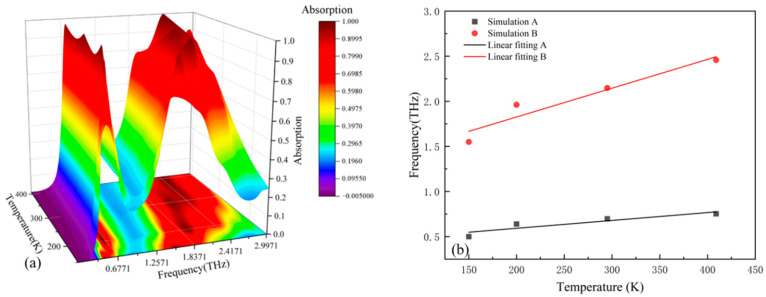
(**a**) Absorption spectra at different temperatures when the sensor is in the narrowband absorption mode. (**b**) Center frequencies and fitted broken lines at different temperatures. Through these two different working modes, more comprehensive temperature information can be obtained, which can, to a certain extent, enhance the accuracy of temperature detection.

To quantify the impact of the device temperature change on the resonant frequency, linear fitting is carried out using the formula $S=\left|\Delta f/\Delta K\right|$ (where S denotes the sensitivity, Δf corresponds to the shift in the device’s resonant frequency, while ΔK indicates the corresponding change in device temperature) [33]. The sensitivities of the first peak A and the second peak B after linear fitting are found to be 2.88 and 3.29 GHz/K respectively. To present the impact of the device temperature on the resonant frequency more intuitively, a fitting graph is plotted, as shown in Figure 12b:

Temperature-dependent spectral responses at 100 meV Fermi energy are presented in Figure 13a. Thermal progression from 150 K to 409 K induces significant displacement in dual-resonance spectral positions: ƒ_1_ undergoes a transition from 0.5002 THz to 0.7525 THz, while ƒ_2_ migrates from 1.550 THz to 2.4577 THz due to strontium titanate’s temperature-sensitive permittivity characteristics.

By applying the formula $S=\left|\Delta f/\Delta K\right|$ to linearly fit the center frequencies, the fitted sensitivities are found to be 0.87518 and 3.19 GHz/K.

**Figure 14:**
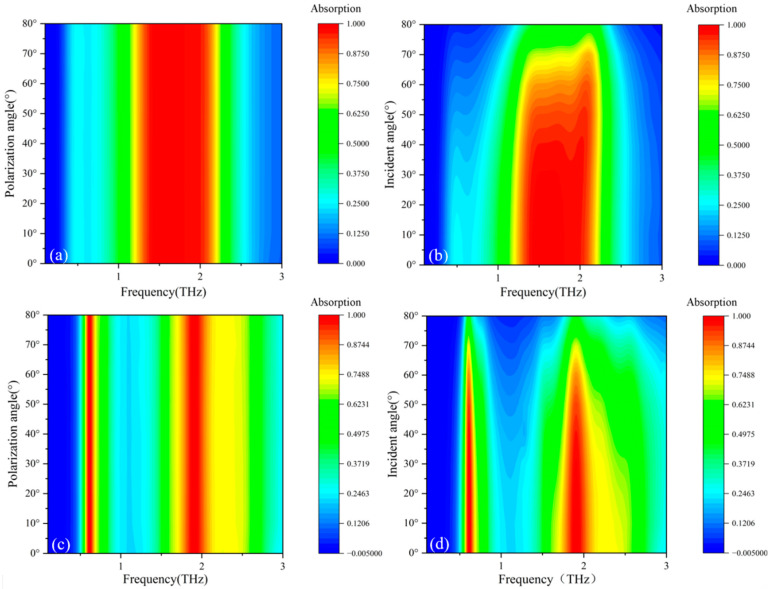
(**a**) Different polarization angles of the device at 35 meV; (**b**) different incident angles of the device at 35 meV; (**c**) different polarization angles of the device at 35 meV; (**d**) different incident angles of the device at 100 meV.

Operational deployment necessitates rigorous analysis of terahertz wave angular dependencies, specifically polarization orientation and incidence variation, on device stability. Figure 14 quantifies absorption efficiency modulation across these parametric conditions.

Figure 14a,b depict the absorption intensities at different polarization angles and incident angles for broadband absorption, while c and d show those for narrowband absorption. As can be seen from Figure 14a,c, when the Fermi levels of the Dirac semimetal are 35 meV and 100 meV, the polarization angle has no significant impact on the absorption efficiency. This polarization insensitivity is generally determined by the symmetry of the structure.

Angular resilience constitutes a critical operational parameter for terahertz absorbers under oblique irradiation. Figure 14b demonstrates exceptional bandwidth stability within 0–60° incidence. Beyond this threshold, progressive absorption attenuation occurs alongside high-frequency spectral displacement. Complementary Figure 14d confirms consistent energy dissipation bandwidth maintenance below 60° incidence, whereas pronounced bandwidth contraction emerges at higher angles. Given inevitable environmental and system-induced variations in irradiation geometry, the device maintains >90% absorption efficiency across wide angular and polarization spectra. This angular tolerance significantly enhances operational robustness in practical deployment scenarios. Table 2 compares the sensitivity of different wave absorbers.

## 4. Conclusions

This study computationally demonstrates a dual-harmonic absorber employing Dirac semimetal and crystalline SrTiO_3_. By leveraging Fermi level tunability in the semimetal and the thermoresponsive behavior of strontium titanate, the device achieves near-ideal absorption spanning both broadband and narrowband regimes.

At 35 meV Fermi energy in the Dirac semimetal layer, this metamaterial operates as a reconfigurable broadband absorber, achieving >90% absorptivity across 1.347–2.1271 THz spectral coverage. Elevating the Fermi level to 100 meV transitions the device into dual-resonance operation, where both narrowband peaks exhibit near-unity absorption exceeding 90% efficiency.

Compared with other traditional structures, this study achieves dual-mode compatibility of narrowband and broadband, featuring high absorptivity and temperature sensitivity. It also exhibits polarization-insensitive performance with absorptivity exceeding 90% when the incident angle is less than 60°. The wide temperature range from 150 K to 409 K covers application scenarios such as low-temperature, ambient-temperature, and medium- and high-temperature conditions, enabling terahertz sensors to meet the diverse application requirements of aerospace, low-temperature physics, industrial automation, biomedical diagnosis, and high-temperature industrial processes. Compared with traditional STO temperature sensors, the temperature detection method proposed in this paper realizes dynamic regulation of the detection frequency band through the synergistic effect with other materials, and the regulation method is more flexible.

## Figures and Tables

**Table 1:** Structural parameter.

Structural Parameter (μm)												
*h*_1_	*h*_2_	*h*_3_	*h*_4_	*P_x_*	*P_y_*	*L*_1_	*L*_2_	*d*_1_	*d*_2_	*d*_3_	*R*_1_	*R*_2_
0.2	17	0.1	0.2	60	60	40	12	5	4	2.5	55	48

**Table 2:** Comparison with published studies.

Literature	Compatible Materials	Absorption Type	Absorptivity	Sensitivity (GHz/K)	Bandwidth	Angular Tolerance	Temperature Range
[15]	STO & Graphene	Narrowband	>99%	0.65		Absorptivity ≥75% at incident angle <70°	200–400 K
[22]	STO & BDS	Narrowband	89–100%	2.20		Absorptivity ≥0.8 at incident angle <70°	150–300 K
[23]	STO & BDS	Broadband	>80% (broadband), 96% & 100% (broadband peaks)	1.29	0.66–0.81 THz (temp.-dependent)	Stable absorption at incident angle ≤40°	250–400 K
This thesis	STO & BDS	Narrowband & Broadband	>90% (broadband), 99.63% & 99.91% (narrowband)	3.19	0.6119–0.8584 THz (temp.-dependent)	Absorptivity >90% when the incident angle ≤60°	150–409 K

## Data Availability

All data that support the findings of this study are included within the article.
